# *Staphylococcus aureus* strains exhibit heterogenous tolerance to direct cold atmospheric plasma therapy

**DOI:** 10.1016/j.bioflm.2023.100123

**Published:** 2023-04-15

**Authors:** Abdullah Baz, Ahmed Bakri, Mark Butcher, Bryn Short, Bhagirath Ghimire, Nishtha Gaur, Toby Jenkins, Robert D. Short, Marcello Riggio, Craig Williams, Gordon Ramage, Jason L. Brown

**Affiliations:** a Oral Sciences Research Group, Glasgow Dental School, School of Medicine, Dentistry & Nursing, College of Medical, Veterinary and Life Sciences, University of Glasgow, Glasgow, G12 8TA, United Kingdom; b Glasgow Biofilm Research Network, 378 Sauchiehall Street, Glasgow, G2 3JZ, United Kingdom; c Department of Chemistry and Material Science Institute, University of Lancaster, Lancaster, LA1 4YB, United Kingdom; d Department of Chemistry, University of Bath, Bath, BA2 7AY, United Kingdom; e Microbiology Department, Lancaster Royal Infirmary, University of Lancaster, Lancaster, LA1 4YW, United Kingdom

**Keywords:** Biofilm, *Candida auris*, *Candida albicans*, *Pseudomonas aeruginosa*, *Staphylococcus aureus*, Heterogeneity, Cold atmospheric plasma, Tolerance

## Abstract

The global clinical and socioeconomic impact of chronic wounds is substantial. The main difficulty that clinicians face during the treatment of chronic wounds is the risk of infection at the wound site. Infected wounds arise from an accumulation of microbial aggregates in the wound bed, leading to the formation of polymicrobial biofilms that can be largely resistant to antibiotic therapy. Therefore, it is essential for studies to identify novel therapeutics to alleviate biofilm infections. One innovative technique is the use of cold atmospheric plasma (CAP) which has been shown to possess promising antimicrobial and immunomodulatory properties. Here, different clinically relevant biofilm models will be treated with cold atmospheric plasma to assess its efficacy and killing effects. Biofilm viability was assessed using live dead qPCR, and morphological changes associated with CAP evaluated using scanning electron microscopy (SEM). Results indicated that CAP was effective against *Candida albicans* and *Pseudomonas aeruginosa*, both as mono-species biofilms and when grown in a triadic model system. CAP also significantly reduced viability in the nosocomial pathogen, *Candida auris*. *Staphylococcus aureus* Newman exhibited a level of tolerance to CAP therapy, both when grown alone or in the triadic model when grown alongside *C. albicans* and *P. aeruginosa*. However, this degree of tolerance exhibited by *S. aureus* was strain dependent. At a microscopic level, biofilm treatment led to subtle changes in morphology in the susceptible biofilms, with evidence of cellular deflation and shrinkage. Taken together, these results indicate a promising application of direct CAP therapy in combatting wound and skin-related biofilm infections, although biofilm composition may affect the treatment efficacy.

## 1 Introduction

Wound infections such as diabetic foot ulcers (DFUs) can be highly problematic in terms of clinical management. They carry a significant socioeconomic burden, costing the NHS an estimated £8.3 billion in 2017/2018 [1], with total costs attached to wound care and management worldwide predicted at upwards of ∼$100 billion [2]. Insufficient treatment is largely due to microbial colonization of the wound interfering with sufficient healing and repair of the damaged tissue. It is estimated that between 40% and 60% of DFUs are infected with microbial biofilms [[3], [4], [5], [6]]. These chronic wounds are in general colonized with a variety of aerobic and anaerobic bacterial species [7], in addition to fungi [[8], [9], [10]]. Due to this, there is an obvious and necessary requirement for development of more effective therapies to combat these infections. Indeed, the global advanced wound care market is projected to reach almost ∼$20 billion by 2027 [11].

Due to the increasing threat of antimicrobial resistance (AMR), alternative methodologies for treating infected chronic wounds are paramount. To this end, cold atmospheric plasma (CAP) has arisen as a potential therapy due to its antimicrobial activity, whilst also contributing to wound healing and repair [12]. The mechanism of action is not fully understood, though the antimicrobial effects of CAP is thought to originate via the production of reactive oxygen and nitrogen species [[13], [14], [15]]. One particular species generated by CAP, H_2_O_2_, is widely used as an antiseptic, for topical treatment of infected chronic wounds, therefore its applicability in wound healthcare is pertinent. Promising outcomes of CAP have been demonstrated, with several recent studies establishing its efficacy against planktonic microorganisms and testing its anti-biofilm activity [14,[16], [17], [18]]. In regards to skin and wounds, a number of studies have focused on singular bacteria, such as *Pseudomonas aeruginosa* and *Staphylococcus aureus* [[19], [20], [21], [22], [23], [24]], whilst CAP has also been shown to possess inhibition effects against fungi such as dermatophytes [25] and *C. albicans* [13,15,26]. At a mixed species level, there is evidence of CAP efficacy against a biofilm containing *S. aureus* and *C. albicans* [27], and *S. aureus* with *P. aeruginosa* and *Enterococcus faecalis* [28].

Wound infections are often interkingdom in nature, and as such the consideration of fungi is clinically important; 80% of non-healing DFUs contain fungi, with *C. albicans* being one of the most important species [10]. On this point, we have previously described a triadic *C. albicans* inclusive model which has been shown to differentially respond to antibiotic therapy, with only the combination of antibacterial and antifungal agents capable of reducing consortia viability [29]. A further mycological consideration is *Candida auris*, a deadly nosocomial pathogen with broad levels of antifungal resistance. It can be found alongside commensal bacterial and fungal microflora on the skin [30]. Since its emergence in 2009, *C. auris* outbreaks have been reported in several countries globally [[30], [31], [32]]. The ability of *C. auris* to readily form biofilms on biotic and abiotic surfaces is challenging for hospital units to fully eradicate with antiseptic washes. Indeed, *C. auris* biofilms are much more tolerant to antiseptic washes such as H_2_O_2_ and chlorhexidine (CHX) than *C. albicans*, suggestive it has the potential to survive for longer on abiotic surfaces [33].

The purpose of this study is to assess the potential use of CAP in treating skin and wound-relevant biofilm models as alternative or augmentative therapy for chronic wounds. This study aims to build upon existing literature reporting CAP therapy as a means to eradicate complex biofilms, by testing its activity against simple mono-species models relating to wound-related microorganisms, a polymicrobial consortia and the nosocomial pathogen, *C. auris*.

## 2 Materials and methods

### 2.1 Microbial growth and standardization

All isolates were stored long-term on Microbank beads at −80 °C prior to revival. The following strains were used for this study; *C. albicans* SC5314, *S. aureus* Newman (ATCC 25904), *S. aureus* NCTC 6571, *S. aureus* SH1000, *S. aureus* ATCC 25923, *P. aeruginosa* PA14, *C. auris* NCPF 8973 and *C. auris* NCPF 8978. The two *C. auris* strains were selected to represent the non-aggregating and aggregating phenotype respectively [34]. Bacterial strains were revived on Luria Bertoni (LB) agar for 24 h at 37 °C, then sub-cultured into 10 mL of LB overnight at 37 °C in a shaker incubator at 200 rpm. For all three fungal strains, these were inoculated onto Sabouraud's dextrose agar (SAB) and incubated at 30 °C for 48 h. Cells were then propagated into yeast peptone dextrose media (YPD) at 30 °C for 18 h in a shaker incubator at 200 rpm. Subsequently, cells were centrifuged at 3000 rpm for 5 min before washing with phosphate buffered saline (PBS) twice before standardization. All *S. aureus* strains and *P. aeruginosa* were standardized to 1 × 10^8^ colony forming equivalent (CFU) per mL using a spectrophotometer; the OD value and wavelength used was 0.6 at OD_600nm_. Yeast cells were diluted 1:100 then counted using a Neubauer haemocytometer.

### 2.2 Biofilm formation

All mono-species and triadic biofilms were grown using a hydrogel model, as we previously described [29,35]. Briefly, each hydrogel contained 10% 3-sulfopropyl acrylate potassium salt, 0.95% polyethylene glycol deacrylate and 0.01% 1-hydroxy cyclohexyl phenyl ketone dissolved in 2X PBS. An equal volume of heat-inactivated horse serum was added to the mixture before transferring 1 mL to 12- well plates. Hydrogels were set following exposure to 365 nm ultraviolet light for 1 h. All standardised isolates (final concentration of 1 × 10^6^ cells/mL in sterile PBS) were allowed to attach to sterile cellulose matrix (1.25 cm^2^ by 1.25 cm^2^) for 2 h at 37 °C with agitation at 180 rpm. Each matrix was washed once in PBS to remove non-adherent cells and then placed onto a hydrogel and incubated at 37 °C for 24 h.

### 2.3 Cold atmospheric plasma treatment

**Fig. 1:**
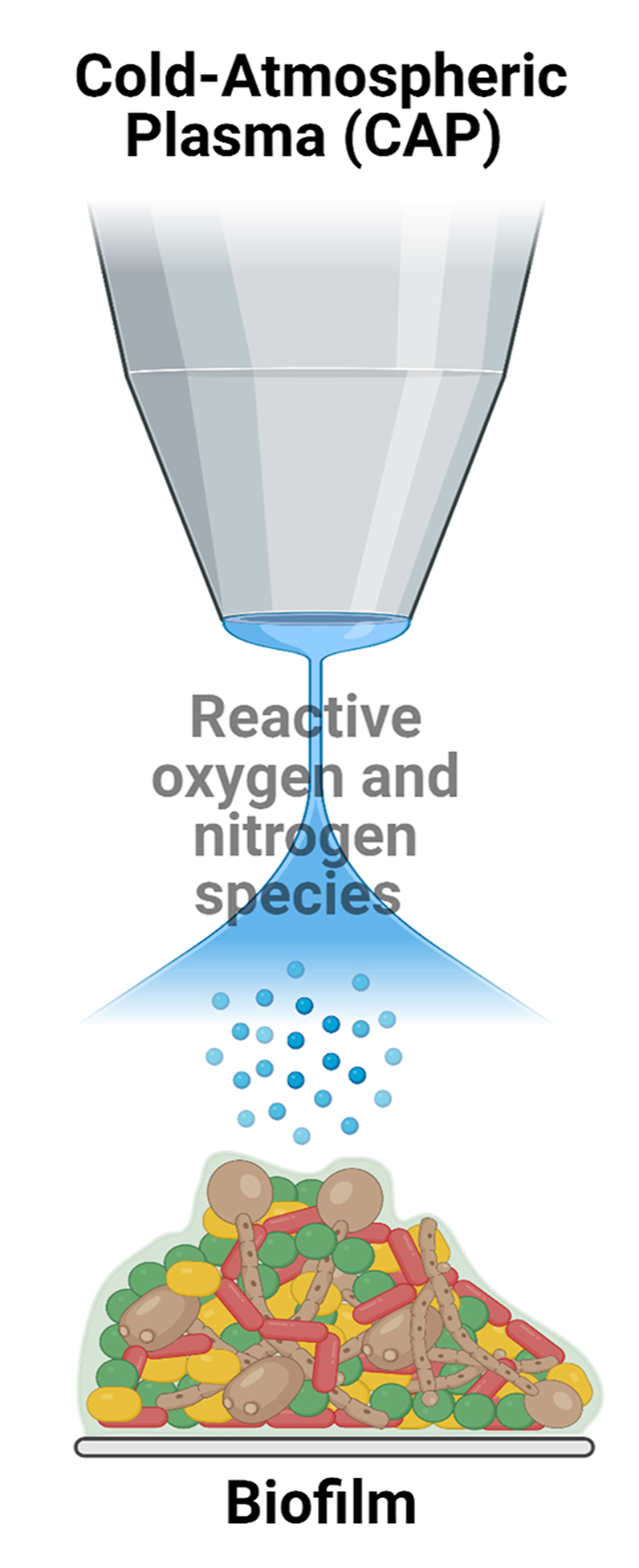
**– Schematic diagram depicting the cold atmospheric plasma therapy of the biofilm models in this study.** The CAP therapy generates different reactive oxygen and nitrogen species such as NO_2_^−^ and H_2_O_2_. A total of eight mono-species biofilms and a triadic mixed-species model were treated with CAP for 5 min within this study. Image created at Biorender.com.

Following biofilm formation, each matrix was washed with PBS to remove non-adherent cells before exposure to CAP for 1, 3 and 5 min. The experimental set-up for the plasma treatment was as described elsewhere [36] and schematized in Fig. 1. In brief, the CAP device itself consisted of six plasma jets arranged in a rectangular pattern. Cold atmospheric plasma (CAP) was generated with pure argon gas (99.9999% purity) through each quartz tube at an applied voltage of 8 kV p–p (peak-peak) and a frequency of 23.5 kHz. The flow rate of gas was set at six standard litres per minute. The biofilms were treated with a 1 cm distance between the end of the quartz tube and the surface of the biofilm-containing cellulose matrix. Due to the porous nature of the cellulose matrix, the material was inverted half-way through each treatment to ensure sufficient CAP coverage of both sides of the matrix. Appropriate argon only controls were initially tested to ensure for CAP-only activity against the biofilms. Following treatment, viability of biofilms was assessed by quantifying colony forming equivalents (CFE) using quantitative PCR and visualised using SEM.

### 2.4 Assessment of biofilm viability

To assess biofilm viability following exposure to CAP, biofilm biomass was removed from cellulose matrices via sonication in 1 mL of sterile PBS at 35 kHz in an ultrasonic water bath for 10 min. Following sonication, 500 μL of biofilm sonicate was exposed to propidium monoazide (PMA) to allow for live/dead qPCR. The remaining 500 μL was used as a control minus PMA. PMA treatment involved a 10 min incubation in the dark to allow cellular uptake of the dye and subsequent exposure to a 650 W halogen light for 5 min. Samples were stored at −20 °C before DNA extraction using the MasterPure Yeast DNA purification kit or QIAamp DNA Mini Kit, both according to manufacturer's instructions. For the latter, an additional lysis step was included. This involved mechanical disruption of the sonicate using 0.5 mm glass beads and homogenisation for 90 s using BeadBug™ microtube homogeniser. Real time qPCR with 18S or *C. auris*-specific and bacterial species-specific primers was performed as previously described [29,37] and CFE/mL was calculated using a standard curve for each organism and/or strain. Standard curves were generated by extracting DNA from pure culture of each species standardized to 1 × 10^8^ CFU/mL, and serially diluting to 1 × 10^3^ CFU/mL. Total (-PMA) and viable (+PMA) CFEs were utilised to determine biofilm viability, expressed as a percentage. For some experiments CFE analysis allowed for assessing % composition of the triadic model pre- and post-regrowth.

### 2.5 Scanning electron microscopy

Biofilm morphology was visualised using the JEOL JSM-6400 SEM machine (JEOL Ltd, Hertfordshire, UK) at magnification of × 1500 for fungal biofilms, or × 3500 for bacterial mono-species and triadic model biofilms. Biofilms were processed and prepared for SEM as previously described [38].

### 2.6 Regrowth assessment

For regrowth of the triadic model, 24-h biofilms were treated as above, then placed back on a fresh hydrogel for another 24 h. The cellulose matrix was re-hydrated with 200 μL of sterile ddH_2_O and placed back in the 37 °C incubator. Following regrowth, biofilms were then processed as above for DNA extraction, live/dead qPCR and composition analysis.

### 2.7 H_2_O_2_ quantification

To assess levels of H_2_O_2_ created from the plasma therapy, plasma activated water (PAW) was generated in a similar manner as previously discussed [20]. In brief, 1.5 mL of sterile ddH2O was added to 12-well plates then exposed to CAP for 1, 3 and 5 min. Following this, H_2_O_2_ was quantified using the Amplex™ Red Hydrogen Peroxide/Peroxidase Assay Kit (Thermo-Fisher, UK) as per manufacturer's instructions, with concentrations determined from a H_2_O_2_ standard curve utilised per assay run.

### 2.8 Statistical analysis

All graph production and data analysis were performed using GraphPad Prism version 9 (La Jolla, California, USA). The Shapiro-Wilk test was utilised to assess the raw CFE/mL data for normality, and after confirming that all data was non-normally distributed, the Mann-Whitney test was then used to make comparisons between CAP-treated and untreated biofilms. Statistical differences were achieved if *p < 0.05. All biofilm data presented here were based on three technical replicates from three biological repeated experiments (n = 9).

## 3 Results

**Fig. 2:**
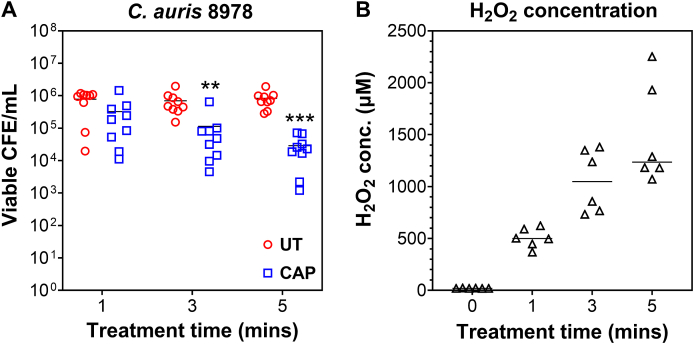
**– Time dependent killing effect of cold atmospheric plasma correlates with increasing concentrations of H**_**2**_**O**_**2**_**.** (**A**) *Candida auris* 8978 biofilms were grown for 24 h within the cellulose matrix-hydrogel system. Following washing with PBS, biofilms were treated for 1, 3 and 5 min with cold atmospheric plasma (CAP) then viability was assessed via live/dead qPCR using colony forming equivalents/mL (CFE/mL). Untreated (UT) controls received no CAP therapy. (**B**) To generate plasma activated water (PAW), sterile ddH_2_O was treated with CAP for 1, 3 and 5 min and H_2_O_2_ produced was quantified using the Invitrogen™ Amplex™ Red Hydrogen Peroxide/Peroxidase Assay Kit according to manufacturer's instructions. Untreated water minus CAP therapy was used for comparison (as denoted by 0 min). Biofilm experiments were completed on three separate occasions with three technical replicates per experiment (n = 9 in total). H_2_O_2_ levels were determined from 6 independently CAP-treated PAW using a standard curve of known H_2_O_2_ concentrations. In panel A, the Mann-Whitney test was used to compare the CFE/mL means of CAP-treated biofilms with their appropriate controls at each time point (**p < 0.01 and ***p < 0.001). (For interpretation of the references to colour in this figure legend, the reader is referred to the Web version of this article.)

It was firstly deemed pertinent to assess the optimal time for killing of the CAP therapy. To do this, *C. auris* NCPF 8978 was selected to demonstrate the effects of CAP following 1, 3 and 5 min treatment. 24-hour biofilms of *C. auris* were treated with CAP, then viable CFE counts quantified using live/dead qPCR. Fig. 2A shows that CAP exhibited a time-dependent killing of *C. auris*, with the greatest effect seen at 5 min treatment time. There was no significant differences in viable cell counts at 1 min (∼7.83 × 10^5^ CFE/mL in untreated vs ∼ 3.24 × 10^5^ CFE/mL in treated). At both 3 and 5 min treatment, significant changes in viability were observed, with a 6.2 fold and 28.9 fold reduction in viable cells following treatment (**p < 0.01 and ***p < 0.001, respectively). In an attempt to propose a mechanism of action for CAP killing effects, concentrations of H_2_O_2_ were detected following exposure of CAP to water (Fig. 2B). Treatment of water for 1, 3 and 5 min CAP activity highlighted a dose dependent increase in H_2_O_2_ levels in the plasma activated water (PAW), ranging from approx. 500 μM at 1 min exposure to almost 1500 μM following 5 min of treatment. Although other reactive oxygen and nitrogen species were not investigated, we propose that CAP killing effects were likely due to increasing H_2_O_2_ levels, which is line with previous observations for this and similar CAP devices [20,36].

**Fig. 3:**
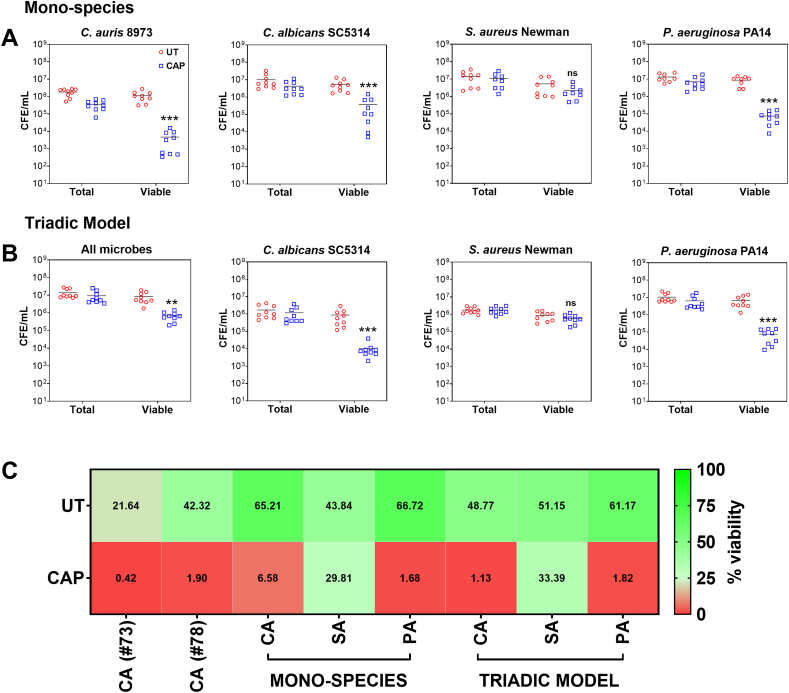
**– *Staphylococcus aureus* Newman displays tolerance traits to cold atmospheric plasma therapy in a mono-species and triadic biofilm model.** (**A**) *Candida auris* 8973, *Candida albicans* SC5314, *Staphylococcus aureus* Newman and *Pseudomonas aeruginosa* PA14 biofilms were formed for 24 h then treated with cold atmospheric plasma (CAP) for 5 min. Total and viable colony forming equivalents/mL (CFE/mL) was quantified for each biofilm following treatment using live/dead qPCR. (**B**) A triadic polymicrobial biofilm model of *C. albicans* SC5314, *S. aureus* Newman and *P. aeruginosa* PA14 was created and treated with CAP in the same manner as above. The CFE/mL counts for all combined microorganisms and for each individual microorganism are shown. (**C**) The heatmap depicts the % viability for each microorganism when grown as mono-species biofilms, or for the triadic biofilm model. Biofilm experiments were completed on three separate occasions with three technical replicates per experiment (n = 9 in total). In panels A and B, the Mann-Whitney test was used to compare the viable CFE/mL means of CAP-treated biofilms with untreated biofilms (**p < 0.01 and ***p < 0.001). No significant differences were observed in the total CFE/mL counts for any microorganism.

Once it was confirmed that 5 min of CAP treatment gave the greatest reductions in viability, CAP was tested on five other biofilm models, including *C. auris* NCPF 8973, *C. albicans* SC5314, *S. aureus* Newman, *P. aeruginosa* PA14 and a triadic model containing the latter three microorganisms. Fig. 3A highlights that CAP therapy was effective against all microorganisms in biofilms, with the exception of *S. aureus* which exhibited a level of tolerance to the treatment. Significant reductions in viable counts were observed in mono-species biofilms of *C. auris* NCPF 8973 (***p < 0.001), *C. albicans* (***p < 0.001) and *P. aeruginosa* (***p < 0.001) (Fig. 2A). Similar observations were seen for viable counts in the triadic model for *C. albicans* and *P. aeruginosa* only (both ***p < 0.001) (Fig. 3B). No changes in viable counts were seen for *S. aureus* grown either alone (∼5.29 × 10^6^ CFE/mL for untreated compared to ∼2.22 × 10^6^ CFE/mL, respectively) or in the multi-species biofilm model (∼8.70 × 10^5^ CFE/mL vs 5.92 × 10^5^ CFE/mL, respectively). To verify that total counts were not affected following CAP therapy, the total CFE/mL counts were included in Fig. 3. No differences were observed in the total counts of cells in the biofilms following CAP treatment, suggesting that CAP had a bacterio- or fungicidal effects rather than biofilm disruption and dispersal-like effects. These total CFE/mL counts were finally utilised for assessing the % viability of untreated and treated biofilms for all microorganisms including *C. auris* NCPF 8978 (Fig. 3C). In line with the above observations, CAP therapy reduced the % viability of all mono- and mixed-species biofilms when compared to the untreated controls, except for *S. aureus* which was tolerant to CAP therapy.

**Fig. 4:**
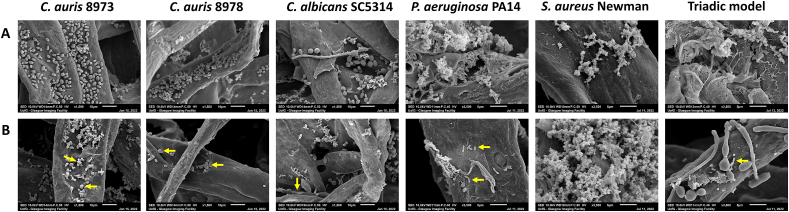
**– Scanning electron microscopy images highlights morphological changes at a cellular level following cold atmospheric plasma therapy.** Untreated or cold atmospheric plasma (CAP)-treated biofilms of *Candida auris* 8973, 8978, *Candida albicans* SC5314, *Staphylococcus aureus* Newman and *Pseudomonas aeruginosa* PA14 were visualised using scanning electron microscopy (SEM). A triadic polymicrobial biofilm model of *C. albicans* SC5314, *S. aureus* Newman and *P. aeruginosa* PA14 was also viewed microscopically following treatment. All biofilms were formed for 24 h, then left untreated (**A**) or treated with CAP for 5 min (**B**), prior to processing for SEM. Yellow arrows denote changes in morphological structures, with evidence of cellular deflation and/or shrinkage. (For interpretation of the references to colour in this figure legend, the reader is referred to the Web version of this article.)

At the microscopic level, there was evidence of morphological changes in CAP-treated biofilms following exposure for 5 min (Fig. 4). In the untreated controls of *C. auris*, there was evidence of oval-shaped yeast cells, with single cell phenotype in the NCPF 8973 strain and clustered aggregates in the NCPF 8978 strain. Conversely, the treated biofilms contained cells with altered morphologies, with evidence of deflation, wrinkling of the cellular structure and disruption to cell integrity. For *C. albicans* mono-species, yeast cells and hyphae were present in the control biofilms, whilst the CAP-treated biofilms possessed hyphae which was deflated resulting in a subtle loss of shape. *P. aeruginosa* mono-species and triadic CAP-treated biofilms appeared to have less extracellular matrix than control biofilms. No obvious changes were seen in the *S. aureus* Newman biofilms, with evidence of dense extracellular matrix in both untreated and CAP-treated biofilms, encapsulating the clusters of cocci-shaped cells.

To assess whether the level of tolerance to CAP was unique to *S. aureus* Newman only, three additional strains of the microorganism were selected for plasma testing; NCTC 6571, SH1000 and ATCC 25923. The total and viable counts for these new strains tested are shown in Supplementary Fig. 1 for comparative purposes alongside previous results for *S. aureus* Newman (Supplementary Figs. 1A and 1B). *S. aureus* NCTC 6571 and SH1000 were largely resistant to CAP therapy, with no statistical differences observed between the viable counts in treated and untreated samples (Supplementary Figs. 1C and 1D). Of the three alternative strains tested, significant reductions in viable cell counts were only observed in the ATCC 25923 strain which resulted in approximately a ∼1-log_10_ reduction in viable counts (4.81 × 10^6^ CFE/mL for untreated compared to 3.05 × 10^5^ CFE/mL for treated, *p < 0.05) (Supplementary Fig. 1E). Although statistical differences were not observed between the viable counts, CAP treatment did reduce the overall % viability of the NCTC 6571 biofilm from ∼24.97% to ∼7.82% (Supplementary Fig. 1F). These results are suggestive that *S. aureus* susceptibility to CAP treatment is strain dependent.

**Fig. 5:**
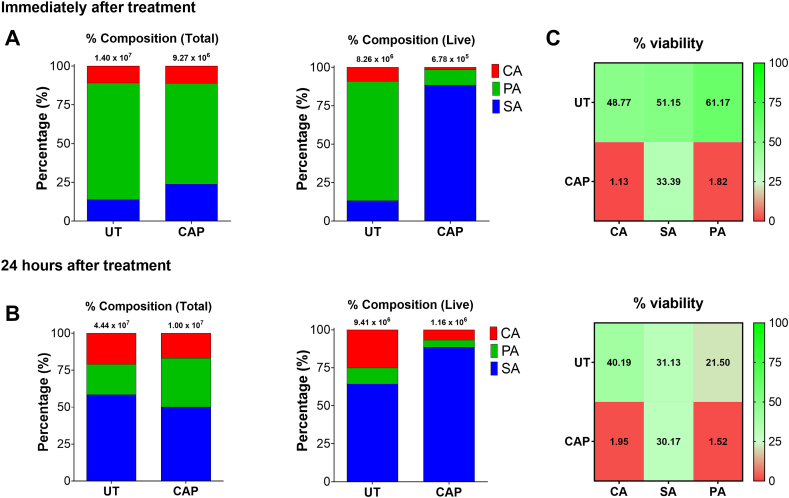
**– Compositional changes in the triadic biofilm model following treatment and biofilm regrowth.** The triadic model containing *Candida albicans* SC5314, *Staphylococcus aureus* Newman and *Pseudomonas aeruginosa* PA14 was matured for 24 h then treated with cold atmospheric plasma. Either, immediately after treatment (**A**), or following regrowth for 24 h (**B**), the total and viable composition of the biofilm was determined using live/dead quantitative PCR. The heatmap depicts the % viability immediately after treatment (results taken from Fig. 2 for comparison) and following regrowth (**C**). Results representative of mean values from n = 9 (three technical replicates from three biological experimental repeats).

Finally to assess further whether the CAP therapy would prevent regrowth of microorganisms, the triadic model biofilms were re-cultured following treatment (Fig. 5). Results indicated that untreated and treated biofilms had similar regrowth patterns after 24 h, with the total viable counts for the untreated biofilms changing from ∼8.26 × 10^6^ CFE/mL to ∼9.41 × 10^6^ CFE/mL. Conversely, the treated biofilms changed from ∼6.78 × 10^5^ CFE/mL immediately after CAP therapy, to ∼1.16 × 10^6^ CFE/mL. It can be concluded from this that the level of regrowth was comparable between control and treated biofilms, however, the composition of the biofilm did change following regrowth. Immediately after treatment, *S. aureus* Newman was the main viable component of the biofilm (∼88.5%) vs *P. aeruginosa* for the untreated biofilm (∼77.7%). Interestingly, the total biofilm composition (e.g., viable and dead cells) was comparable immediately after treatment and 24 h after regrowth. Following regrowth for 24 h, biofilms were predominated by *S. aureus* Newman regardless of treatment (∼64.3% vs ∼88.6% for untreated and treated biofilms, respectively). The % viability of *S. aureus* Newman was also similar in untreated and treated biofilms after regrowth, whilst *C. albicans* and *P. aeruginosa* viability were comparable to that immediately after treatment (∼1.95% and ∼1.52% vs ∼1.13% and ∼1.82%, respectively; Fig. 5C). As expected there was a disparity in the % viability for these two susceptible microorganisms and *S. aureus* Newman immediately following treatment and after regrowth. Taken together, results in Fig. 3, Fig. 5 suggest that *S. aureus* Newman exhibits a level of tolerance to CAP therapy, although this phenotype is strain specific (Supplementary Fig. 1).

## 4 Discussion

This study has demonstrated the killing effects of CAP against a variety of bacterial, fungal and mixed-species biofilms *in vitro*. It was identified that CAP therapy for 5 min was most effective for reducing biofilm viability compared to 1 and 3 min treatment. CAP activity for 5 min was sufficient in significantly decreasing the viable counts in mono-species biofilms of aggregating and non-aggregating strains of *C. auris*, *C. albicans* and *P. aeruginosa*. *S. aureus* Newman-containing biofilms were tolerant to CAP treatment, when grown alone or in triadic combination with susceptible microorganisms, *C. albicans* and *P. aeruginosa*. Interestingly, this effect was strain dependent, with three other strains of *S. aureus* exhibiting varying degrees of susceptibility to CAP therapy.

To this date, the exact mechanism of CAP activity is not fully understood, although it is proposed that the generation of reactive oxygen and nitrogen species leads to antimicrobial activity against bacteria, fungi and viruses. It is proposed that several short and long-lived reactive species and free radicals are produced by CAP such as superoxide anions (O_2_^−^), hydroxyl radicals (•OH), nitric oxide (NO), peroxynitrite (ONOO^−^), nitrite (NO_2_^−^), nitrate (NO_3_^−^) and H_2_O_2_ [39,40]. Of these, the reactive nitrogen species of NO, ONOO^−^, NO_2_^−^ and NO_3_^−^ have been shown to possess antibacterial properties [[41], [42], [43]]. In particular, NO_2_^−^ has antimicrobial activity against biofilms of *S. aureus* and *P. aeruginosa* [44,45]. One long-lived reactive nitrogen species commonly produced by CAP is H_2_O_2_, which has widely been known to possess potent antimicrobial activities, and is a compound readily used in wound washes, ointment creams and mouth rinses. The activity of H_2_O_2_ against microbial species stems from the interactions of the H_2_O_2_ and subsequent breakdown into •OH radicals with cellular components outside and inside the microbial cells, causing DNA damage and cell death: several studies have shown that sequestration of these •OH radicals can prevent the H_2_O_2_-mediated death of bacterial species [46,47]. Here, we demonstrated there was a time-dependent killing effect with the CAP therapy on the *C. auris* NCPF 8978 biofilm, which correlated with increasing concentrations of H_2_O_2_. Future studies should consider detection of other reactive oxygen and nitrogen species generated by CAP therapy, to truly delineate the mechanism of action for the plasma. At this juncture, a previous study utilising the same CAP device used here, revealed increasing concentrations of NO_2_^−^ in PAW, peaking at 14 μM after 2 min of treatment [36]. It is worth nothing that the concentrations of NO_2_^−^ produced by this device are 1000- fold lower than concentrations previously shown to be effective against biofilms of *S. aureus* and *P. aeruginosa* [44,45], suggestive that NO_2_^−^ may not be as potent as H_2_O_2_ in driving cell death. Nevertheless, a combinational and synergistic antimicrobial effect of all reactive species cannot be ruled out without further investigations.

CAP therapy was ineffective in reducing viability in *S. aureus* Newman, when grown as mono-species biofilms or in a triadic model alongside *C. albicans* and *P. aeruginosa*. Conversely, observations from this study highlighted that this tolerant phenotype exhibited by *S. aureus* Newman was strain dependent; *S. aureus* ATCC 25923 was more susceptible to CAP therapy resulting in a >1-log_10_ in viable cell counts. This result is in line with results from a previous study [27]. Further to this, recent publications have reported similar CAP susceptibility traits to other *S. aureus* strains [21,22] raising the question of strain heterogeneity in *S. aureus*. Indeed, it has been shown elsewhere that *S. aureus* isolates are genetically heterogeneous in nature, an attribute that can influence their infectious state [48,49]. Interestingly, a recent study by Ref. [49] highlighted that antibiotic resistance gene mutations and biofilm forming capabilities can vary amongst isolates [49]. This is also in line with observations for *Candida* spp. such as *C. albicans* and *C. auris*, which have also been shown to exhibit a level of heterogeneity amongst isolates [50,51]. Such genetic and phenotypic variations may influence treatment susceptibility to the reactive species generated by the plasma therapy. If H_2_O_2_ is the main antimicrobial agent produced via CAP, there is evidence in the literature that small colony variants of *S. aureus* may arise following exposure to sub-lethal doses of H_2_O_2_ [52]. In line with this, sub-MIC levels of H_2_O_2_ can lead to enhanced bacterial survival and evolution in genes relating to oxidative stress [53]. Investigating such potential genetic phenomena following regrowth of the CAP-treated biofilms would be of interest, but goes far beyond the scope of the current study. Given that treatment time was only 5 min in this study, an explanation for the observed *S. aureus* tolerance to CAP therapy may simply be that H_2_O_2_ levels were below inhibitory sessile concentrations. Moving forward, assessing CAP efficacy against a variety of laboratory and clinical strains of the microorganisms investigated in this study merits consideration, to truly assess for strain heterogeneity in response to CAP therapy. Such studies should also extend to other clinically relevant Gram positive and Gram negative bacterial species, including anaerobic microorganisms and other fungal pathogens to those studied here. Another possible avenue for future work would be utilising these model systems to study the effects of repeat CAP treatments on biofilm growth. Indeed, this would be in line with recent clinical studies that have described the use of multiple CAP therapies on patients spread across days and/or weeks [54,55].

As discussed briefly above, the mechanism of action of CAP and the observed resistance exhibited by the various *S. aureus* strains is mere postulation. It would be of interest to assess at a molecular level, changes in expression of genes relating to oxidative stress pathways in the strains pre- and post-treatment. It is well documented that *S. aureus* has evolved unique protective and repair pathways to circumvent both endogenous and exogenous oxidative stress [56]. However, *P. aeruginosa* also possesses similar oxidative stress pathways [57]. Therefore, it may be likely that variations in CAP susceptibility between *S. aureus* and *P. aeruginosa* has arose from differences in cell wall structure. Evidence suggests that a peptidoglycan-rich, thicker cell wall provides Gram positive microorganisms with greater protection to plasma activity [58,59]. Alternatively, the thicker extracellular matrix formed by the *S. aureus* Newman biofilms as depicted in the SEM images could be a possible explanation as to the tolerance exhibited by the microorganism. To this end, previous studies have highlighted that biofilm matrix provides an additional physical and chemical barrier to the CAP [60,61]. Regarding the *Candida* spp. used in this study, *C. albicans* biofilms grown in the same hydrogel system have been shown to be susceptible to H_2_O_2_ activity, albeit at much higher concentrations (3% w/v or ∼880 mM) [62]. Interestingly, in the same study H_2_O_2_ was largely ineffective against *C. auris* biofilms following 5 min treatment, only resulting in a ∼1-log_10_ reduction in viable cells. Therefore, the observations that CAP was successful in reducing the viability of both *C. auris* biofilms by ∼2-log_10_ are promising (particularly at concentrations of ∼1500 μM), but also highlights that the antimicrobial activity of CAP may be a combination of H_2_O_2_ with other reactive species generated via the device.

To conclude, this study emphasizes the efficacy of CAP against different biofilm model systems *in vitro*, suggestive that direct plasma treatment could alleviate biofilm-related wound infections. Further work is required to assess the safety of the CAP device, and whether direct treatment of infected skin tissue is feasible both *in vitro* and *in vivo*. Indeed, it may be necessary that •OH radicals generated by the CAP are quenched in order to prevent DNA damage to the host but still have its intended antimicrobial effects [63]. *In vivo* studies investigating CAP activity in treatment of mono-infections have shown limited success [64,65], although the wound healing properties of CAP remain promising in rat models [66,67]. To the authors knowledge, limited model systems exist that have studied both the antimicrobial activity of CAP and the host response within an organotypic chronic wound model in tandem, which would provide an interesting proposition for future work. Such an investigation is necessary to assess the effects of this or similar CAP devices on mammalian cell lines, both in single cell monolayers or in 3D-tissue models. Nevertheless, several clinical trials have recently shown favourable results with regards to CAP accelerating wound healing in patients with DFUs or skin grafts, emphasizing the beneficial immunomodulatory properties of the therapy [55,68,69]. Everything considered, CAP clearly appears to possess antimicrobial properties, particularly against biofilms, meaning this therapy is a potential alternative to antibiotics in treating infected chronic wounds.

## Author contributions

JLB participated in investigation, analysis and interpretation of data, responsible for preparation of the manuscript, involved in conception and design of the study and supervision. AB (Abdullah Baz) and AB (Ahmed Bakri) participated in investigation, analysis and interpretation of the data and were responsible for preparation of the manuscript. MCB and BS participated in investigation, analysis and interpretation of the data. BG and NG participated in methodology, investigation, and conception and design of the study. TJ, RDS, CW and GR participated in conception and design of the study and were responsible for funding acquisition and supervision. MR participated in conception and design of the study and supervision. All authors contributed to the article and approved the submitted version.

## Declaration of competing interest

The authors would like to declare that they have no competing interests in regards to the manuscript entitled “*Staphylococcus aureus* strains exhibit heterogenous tolerance to direct cold atmospheric plasma therapy”. Co-author Gordon Ramage as editor for Biofilm journal, had no involvement in the peer review of this article and has no access to information regarding its peer review. Full responsibility for the editorial process for this article was delegated to Tom Coenye.

## Data Availability

Data will be made available on request.
