# Supplementary material for: Staphylococcus aureus strains exhibit heterogenous tolerance to direct cold atmospheric plasma therapy
Source: Biofilm. 2023 Apr 15;5:100123. doi: 10.1016/j.bioflm.2023.100123 (PMC10149328; doi:10.1016/j.bioflm.2023.100123)
Supplement: Multimedia component 1 [file mmc1.docx]

Supplementary Files

***Staphylococcus aureus strains exhibit heterogenous tolerance to direct cold atmospheric plasma therapy***


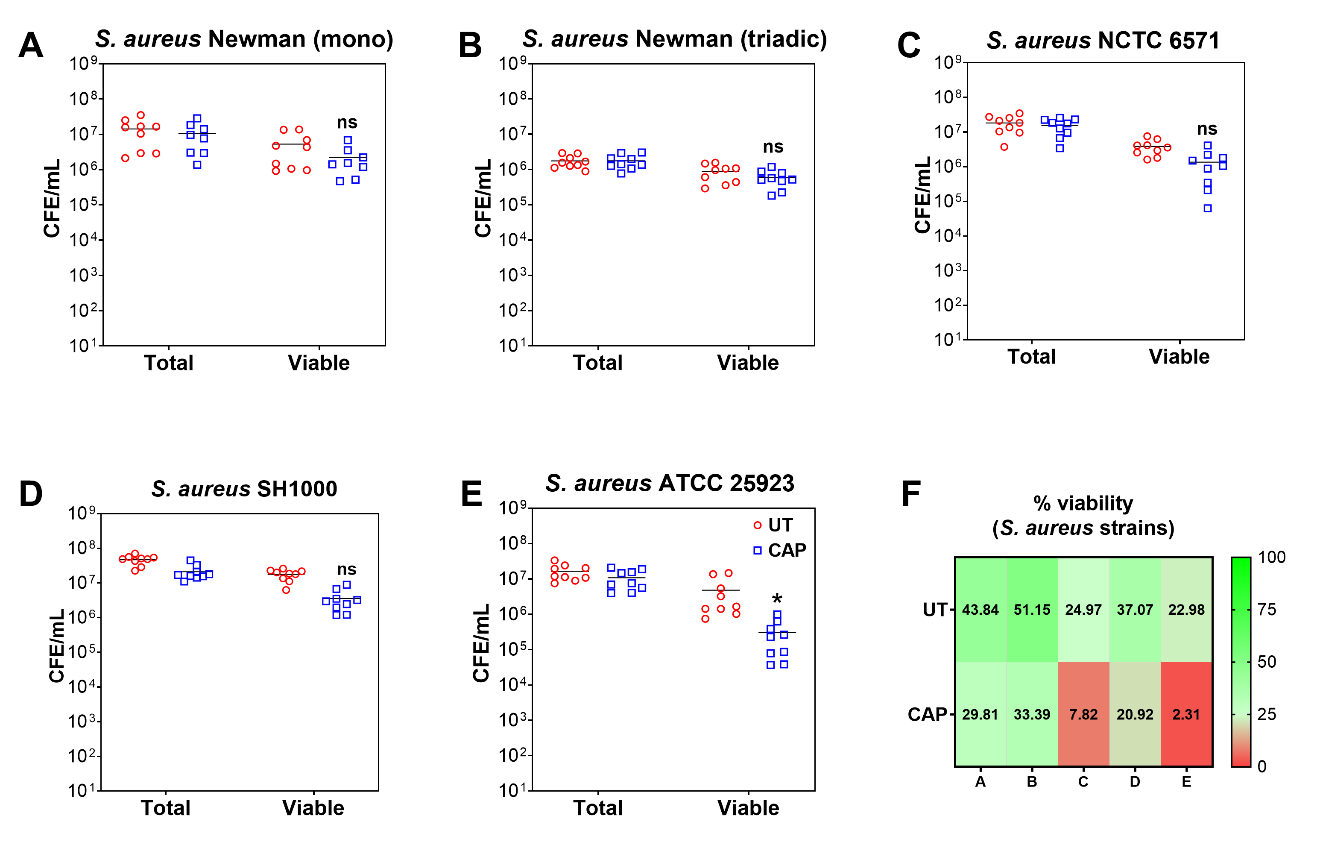


**Supplementary Figure 1 – Biofilms formed from a variety of different *Staphylococcus aureus* strains were assessed for cold atmospheric plasma susceptibility.** Biofilms from four *S. aureus* strains were matured for 24 h in the hydrogel cellulose matrix system, prior to treatment for 5 mins with cold atmospheric plasma therapy. These biofilm models were as follows; *S. aureus* Newman grown alone (A) or in the triadic biofilm model (B), NCTC 6571 (C), SH1000 (D) and ATCC 25923 (E) all as mono-species biofilms. Total and viable colony forming equivalents/mL (CFE/mL) was quantified for each biofilm following treatment using live/dead qPCR. The % viability for all strains are shown in the heatmap (F). Biofilm experiments were completed on three separate occasions with three technical replicates per experiment (n=9 in total). In panels A to E, the Mann-Whitney test was used to compare the viable CFE/mL means of CAP-treated biofilms with untreated biofilms (* p<0.05). ns refers to no significant differences between the viable counts.
